# Dosimetry of Various Human Bodies Exposed to Microwave Broadband Electromagnetic Pulses

**DOI:** 10.3389/fpubh.2021.725310

**Published:** 2021-08-13

**Authors:** Jerdvisanop Chakarothai, Kanako Wake, Katsumi Fujii

**Affiliations:** Electromagnetic Compatibility Laboratory, Electromagnetic Standards Research Center, Radio Research Institute, National Institute of Information and Communications Technology, Koganei, Japan

**Keywords:** electromagnetic pulses, finite-difference time-domain method, fast inverse Laplace transform, Prony method, exposure assessment

## Abstract

In this paper, human exposures to ultra-wideband (UWB) electromagnetic (EM) pulses in the microwave region are assessed using a frequency-dependent FDTD scheme previously proposed by the authors. Complex permittivity functions of all biological tissues used in the numerical analyses are accurately expressed by the four-term Cole–Cole model. In our method, we apply the fast inverse Laplace transform to determine the time-domain impulse response, utilize the Prony method to find the *Z*-domain representation, and extract residues and poles for use in the FDTD formulation. Update equations for the electric field are then derived via the *Z*-transformation. Firstly, we perform reflection and transmission analyses of a multilayer composed of six different biological tissues and then confirm the validity of the proposed method by comparison with analytical results. Finally, numerical dosimetry of various human bodies exposed to EM pulses from the front in the microwave frequency range is performed, and the specific energy absorption is evaluated and compared with that prescribed in international guidelines.

## Introduction

In 2002, the Federal Communications Commission of the United States of America issued a ruling allowing the use of ultra-wideband (UWB) electromagnetic (EM) pulses in the frequency range between 3.1 and 10.6 GHz (1). Since then, numerous applications of UWB pulses have emerged as a result of the regulation, such as vital signal detection and locating moving human bodies, as well as ground-penetrating radar, remote sensing, non-destructive inspection, and so forth (2). Owing to many advantages of UWB pulses such as low power consumption and immunity to multipath of EM propagations and EM interferences, the widespread use of EM pulses is expected to continue. Some of these applications use UWB pulses in the vicinity of human bodies, such as wireless capsule endoscopy using broadband EM pulses for on/in-body communications (3, 4). These applications have led to public concern about the effect on the broadband EM pulses to human body.

Meanwhile, biological effects due to EM pulses have been numerically and experimentally investigated. The effects include the microwave hearing effect, microwave heating effect, and electroporation (5–9). Consequently, international organizations have prescribed exposure limits for the temporal peak of specific energy absorption (SA) in published guidelines to prevent adverse effects, particularly of microwave hearing, which is considered an acute biological effect (10, 11). The International Commission on Non-ionizing Radio Protection (ICNIRP) provided an SA limit of 2 mJ/kg in an arbitrary 10 g-averaged tissue for a single pulse illumination (10), while the SA limit is up to 576 J/kg for continuous exposure of 6 min in the regulation defined by the Institute of Electrical and Electronics Engineers (IEEE) (11). Recently, the ICNIRP has revised the Guidelines based on the relevant scientific knowledge and published them in 2020. In the guidelines, it is mentioned that there is no evidence that microwave hearing in any realistic exposure scenarios causes adverse health consequence and the microwave hearing effect is not considered in the guideline but it is still mandate to consider the heating effect from the pulse exposures (12). Although many biological effects due to EM pulses have been experimentally confirmed, there are few studies providing detailed exposure levels or showing the distribution of SA inside a human body.

To derive SA inside the human body, calculation of the interactions between EM pulses and biological bodies is necessary. In the earliest studies, most of the biological targets were objects having simple shapes such as a multilayer or a dielectric sphere, inside which SA or the induced electric field was derived theoretically (13, 14). However, there has been no detailed dosimetric information of the detailed human body exposed to EM pulses due to difficulties in the calculation of SA or the induced electric field inside biological bodies. These problems are mainly attributed to the frequency dependence of the dielectric properties of biological tissues, which are expressed by the four-term Cole-Cole model (15).

To perform numerical dosimetry of EM pulses, we need to consider the frequency dependence of the permittivity and conductivity of biological tissues over a broad frequency range. Many frequency-dependent finite-difference time-domain [(FD)^2^TD] approaches have been proposed, such as recursive convolution method (16), piecewise linear recursive convolution method (17), trapezoidal recursive convolution method (18), auxiliary differential equation method (19–21), and *Z*-transform method (22). However, these approaches have only been applied to materials having complex permittivity expressed by relatively simple models such as the Debye and Lorentz models. These approaches are not applicable to the Cole–Cole function, due to difficulties in finding the exact time-domain solution of a fractional-order differential equation. Nevertheless, many attempts have been made to address this problem, including those using the Riemann–Liouville theory to find the time-domain solution of the model (23, 24). Recently, our research group has proposed an FDTD formulation for analyses of arbitrary frequency-dependent materials via the use of the fast inverse Laplace transform (FILT) and the Prony method (25). The proposed method has also been extended to three-dimensional analyses of UWB antennas in the vicinity of the human body and the dosimetry of EM pulses incident to a human head (26, 27).

In this study, we extend our numerical models to whole-body human models which are exposed to broadband EM pulses. To the best of our knowledge, this is the first ever numerical dosimetry of EM pulses, and detailed information of SA inside the human body is provided for compliance with the SA limit prescribed in international guidelines. The proposed method involves two steps; firstly, we apply the FILT to permittivity functions in the complex frequency domain to transform them into time-domain impulse responses. Then, the Prony method is used to extract the model parameters and to determine expressions for the infinite impulse response (IIR) expressions in the *Z*-domain. The update equations of electric field are derived via the *Z*-transform.

This paper is outlined as follows. The proposed (FD)^2^TD formulation and the calculation of the update coefficients for the electric field are described in section (FD)^2^TD Formulation Using FILT and Prony Method. The validity of the method in calculating SA and internal electric field strength (IEFS) inside a multilayer model of biological tissues is demonstrated via comparison with the theoretical results in section Transmission Characteristics of EM Pulses Into Biological Bodies. Numerical dosimetry of anatomically detailed human body models exposed to UWB EM pulses is performed and physical quantities such as SA and IEFS are quantitatively derived and compared with those prescribed in the guidelines in section Transmission Characteristics of EM Pulses Into Biological Bodies. Finally, conclusions are drawn in section Conclusion.

## (FD)^2^TD Formulation Using FILT and Prony Method

### Methodology

In this study, all media used in the numerical analyses are biological tissues having complex relative permittivity expressed by the four-term Cole–Cole function as

(1) $$\begin{array}{l} \varepsilon_{m}\left(\omega \right)=\varepsilon_{\infty }+\frac{\sigma }{j\omega \varepsilon_{0}}+\overset{4}{\underset{q=1}{\sum }}\frac{\Delta \chi_{q}}{1+{\left(j\omega \tau_{q}\right)}^{1-\alpha_{q}}}, \end{array}$$

where, ω, ε_∞_, and σ are the angular frequency [rad/s], relative permittivity and conductivity [S/m] of a biological medium at infinite frequency, respectively. ε_0_ is the free-space permittivity and Δχ_*q*_ represents the change in relative permittivity in the *q*th relaxation term. τ_*q*_ and α_*q*_ are the relaxation time and a parameter determining the broadness of the *q*th term, respectively. All parameters in (1) can be found in Gabriel's database of dielectric properties for biological tissues (15). Although Gabriel's permittivity data are de facto, it is noteworthy that different Cole–Cole parameters may be derived, depending on the method used in fitting the measurement data of the dielectric properties. By limiting the frequency range to between 1 MHz and 20 GHz, the number of Cole–Cole terms may be reduced from four terms to two terms while providing the best fit to the measurement data (28). The average deviations from the measurement results over a frequency range between 1 MHz and 20 GHz are higher than 20% for both relative permittivity and loss factor of some biological tissues but they are shown to be <15% for the two Cole-Cole terms used in Kensuke et al. (28).

Since the (FD)^2^TD formulation for biological bodies has been described in the literature (26), we hereby only show the numerical procedures of the proposed method and the derived update equation for electric fields. Firstly, we apply the FILT to electric susceptibility represented by the Cole–Cole model, with the relaxation time normalized by the time step interval used in the FDTD simulations and obtain impulse responses for each susceptibility term in the time domain. Then, we use the Prony method to transform the time-domain impulse response into that in the *Z*-domain. The permittivity is now expressed in the *Z*-domain as

(2) $$\begin{array}{l} \varepsilon_{m}\left(z\right)=\varepsilon_{\infty }+\frac{1+z^{-1}}{1-z^{-1}}\frac{\sigma \Delta t}{2\varepsilon_{0}}+\overset{4}{\underset{q=1}{\sum }}\overset{L_{q}}{\underset{l=1}{\sum }}\frac{A_{l}^{\left(q\right)}}{1-p_{l}^{\left(q\right)}z^{-1}}, \end{array}$$

where Δ*t* and *L*_*q*_ are the time step interval and number of poles for the *q*th Cole–Cole term, respectively. $A_{l}^{\left(q\right)}$ and $p_{l}^{\left(q\right)}$ are residues and poles, respectively. Note that the second term of the right hand side is obtained by applying the bilinear approximation, i.e., *jω* ≈ *s* = 2(1 − *z*^−1^)/ (1 + *z*^−1^)/ (Δ*t*). Since the nested summation in the third term of the right-hand side of (2) can be merged into a single summation, (2) can simply be expressed as

(3) $$\begin{array}{l} \varepsilon_{m}\left(z\right)=\varepsilon_{\infty }+\frac{1+z^{-1}}{1-z^{-1}}\frac{\sigma \Delta t}{2\varepsilon_{0}}+\overset{N}{\underset{k=1}{\sum }}\frac{A_{k}}{1-p_{k}z^{-1}}, \end{array}$$

where *N* is the total number of Debye terms, i.e., *N* = *L*_1_ + *L*_2_ + *L*_3_ + *L*_4_. Procedures for determining *N*, *A*_*k*_, and *p*_*k*_ using the Prony method will be described in subsection Calculation of Specific Energy Absorption and Internal Electric Field Strength and can also be found in the literature (25). Substituting (3) into the discrete constitutional relation of Maxwell's equations, we obtain the update equation for the electric field as

(4) $$\begin{array}{l} E^{n}=\frac{1}{L_{0}}\left[\frac{D^{n}}{\varepsilon_{0}}-\frac{\sigma \Delta t}{2\varepsilon_{0}}E^{n-1}-I^{n-1}-\overset{N}{\underset{k=1}{\sum }}p_{k}P_{k}^{n-1}\right], \end{array}$$

where,

(5) $$\begin{array}{lll} L_{0} & = & \varepsilon_{\infty }+\frac{\sigma \Delta t}{2\varepsilon_{0}}+\overset{N}{\underset{k=1}{\sum }}A_{k} \end{array}$$

(6) $$\begin{array}{lll} I^{n} & = & I^{n-1}+\frac{\sigma \Delta t}{2\varepsilon_{0}}\left(E^{n}+E^{n-1}\right) \end{array}$$

(7) $$\begin{array}{lll} P_{k}^{n} & = & p_{k}P_{k}^{n-1}+A_{k}E^{n}, \end{array}$$

**I**^*n*^ and ${\text{P}}_{k}^{n}$ are the auxiliary variables which are initialized by setting **I**^0^ = 0 and ${\text{P}}_{k}^{0}$ = 0, respectively. Equations (4), (6), and (7) are the update equations for the electric field, the auxiliary field used for considering the conductivity term, and the auxiliary field used for considering the Cole–Cole terms, respectively. The update equations for the electric flux density and magnetic field can be obtained by applying the central difference to Maxwell's equations similarly to those in conventional FDTD procedures (20).

The main advantages of the proposed method are that we can avoid the formulation of fractional-order differential equations by using the FILT and the Prony method and it is straightforward to implement the proposed method into the conventional FDTD code.

### Calculation of Specific Energy Absorption and Internal Electric Field Strength

The exposure level inside biological bodies illuminated by broadband EM pulses can be evaluated using SA, which has been used as a metric in the guidelines. In the FDTD simulations, SA can be calculated using the following equation:

(8) $$\begin{array}{l} {\text{SA}}^{n-\frac{1}{2}}=\Delta t\sum_{m=1}^{n}{\;\left(\frac{E\left(t\right)}{\rho }\cdot \frac{\partial D\left(t\right)}{\partial t}\right)|}_{t=\left(m-\frac{1}{2}\right)\Delta t},\\ \;=\frac{1}{2\rho }\sum_{m=1}^{n}\left(E^{m}+E^{m-1}\right)\cdot \left(D^{m}-D^{m-1}\right), \end{array}$$

where ρ is the density of the biological tissue. The electric flux density in Equation (7) at the *n*th time step (**D**^*n*^) is updated using the magnetic field at the (n+1/2)^th^ time step (**H**^*n*+1/2^) and **E**^*n*^ is updated using (4).

Since a broadband pulse is utilized in our simulations, we can also obtain numerical solutions of the electric and magnetic fields at each frequency component within a single run. The electric field at a frequency is determined via Fourier transform of the waveform obtained at an observation location as

(9) $$E\left(\omega \right)=\int_{0}^{T}E\left(t\right)e^{-j\omega t}dt=\sum_{n=0}^{N^{T}}E^{n}e^{-j\omega n\Delta t}\Delta t,$$

where *N*^*T*^ is the total number of time steps. After the electric field at each frequency is obtained, the specific absorption rate (SAR) is then calculated as follows:

(10) $$SAR\left(\omega \right)=\frac{\sigma {\left|E\left(\omega \right)\right|}^{2}}{2\rho }.$$

### Calculation of Update Coefficients for Electric Field

The procedures for determining coefficients *A*_*k*_ and *p*_*k*_ in the update equation for the electric field are described as follows. First, we transform relative permittivity represented in the frequency domain into that in the complex frequency domain by replacing *jω* with the complex frequency *s* and apply the FILT to find impulse response of the permittivity in the time domain. Then, the Prony method is used to extract the residues *A*_*k*_ and poles *p*_*k*_ from the expression for the IIR in the *Z*-domain.

As an example, we apply the FILT and the Prony method to the permittivity functions of biological tissues “Fat” and “Gray Matter.” Each Cole–Cole parameter is taken from the Gabriel's database and listed in Table 1. The time step interval Δ*t* used to normalize the relaxation time in the Cole–Cole function before applying the FILT is set to 1.668 ps.

**Table 1:** Cole–Cole parameters for “Fat” and “Gray Matter” from Gabriel's database.

**Tissue name**	**ε_∞_**	**σ (S/m)**	**1st term**	**2nd term**	**3rd term**	**4th term**
Fat	2.5	0.035	Δχ_1_ = 9 τ_1_ = 7.958 ps α_1_ = 0.2	Δχ_2_ = 35 τ_2_ = 15.915 ns α_2_ = 0.1	Δχ_3_ = 3.3 × 10^4^ τ_3_ = 159.155 ms α_3_ = 0.05	Δχ_4_ = 10^7^ τ_4_ = 15.915 ms α_4_ = 0.01
Gray Matter	4	0.02	Δχ_1_ = 45 τ_1_ = 7.958 ps α_1_ = 0.1	Δχ_2_ = 400 τ_2_ = 15.915 ns α_2_ = 0.15	Δχ_3_ = 2.0 × 10^5^ τ_3_ = 106.103 ms α_3_ = 0.22	Δχ_4_=4.5 × 10^7^ τ_4_ = 5.305 ms α_4_ = 0

Table 2 shows the update coefficients *A*_*k*_ and *p*_*k*_ used in the FDTD calculations for “Fat” and “Gray Matter.” These values are directly obtained from the Prony method and the number of coefficients for each Cole–Cole term is truncated when the ratio of {|*A*_*k*_| /max|*A*_*k*_|} for *k* = 1, 2, …, *N* is less than a tolerance value of 10^−3^. The update coefficients *A*_*k*_ and *p*_*k*_ physically correspond to the initial amplitude and the decreasing ratio of the time-domain impulse response, respectively. From Table 2, the total numbers of coefficients are 15 and 29 for “Fat” and “Gray Matter,” respectively.

**Table 2:** Update coefficients for “Fat” and “Gray Matter.”

**Cole-Cole terms**	**Fat**		**Gray Matter**	
	***A_***k***_***	***p_***k***_***	***A_***k***_***	***p_***k***_***
1st	*A*_1_ = 0.51370 *A*_2_ = 0.44668 *A*_3_ = 0.38560 *A*_4_ = 0.27620 *A*_5_ = 0.18682 *A*_6_ = 0.16287 *A*_7_ = 0.02939 *A*_8_ = 0.00263	*p*_1_ = 0.82495 *p*_2_ = 0.71195 *p*_3_ = 0.54623 *p*_4_ = 0.90559 *p*_5_ = 0.33125 *p*_6_ = 0.96186 *p*_7_ = 0.11328 *p*_8_ = 0.99134	*A*_1_ = 3.94634 *A*_2_ = 2.27434 *A*_3_ = 1.16025 *A*_4_ = 1.01923 *A*_5_ = 0.57702 *A*_6_ = 0.12362 *A*_7_ = 0.32517 *A*_8_ = 0.00886	*p*_1_ = 0.80579 *p*_2_ = 0.69670 *p*_3_ = 0.88246 *p*_4_ = 0.51558 *p*_5_ = 0.29143 *p*_6_ = 0.95064 *p*_7_ = 0.09047 *p*_8_ = 0.98824
2nd	*A*_9_ = 0.00456 *A*_10_ = 0.00137 *A*_11_ = 0.00090 *A*_12_ = 0.00067 *A*_13_ = 0.00054	*p*_9_ = 0.99981 *p*_10_ = 0.99470 *p*_11_ = 0.96716 *p*_12_ = 0.89813 *p*_13_ = 0.77398	*A*_9_ = 0.05766 *A*_10_ = 0.02718 *A*_11_ = 0.01975 *A*_12_ = 0.01582 *A*_13_ = 0.01333 *A*_14_ = 0.01158 *A*_15_ = 0.00997 *A*_16_ = 0.00762	*p*_9_ = 0.99977 *p*_10_ = 0.99451 *p*_11_ = 0.96773 *p*_12_ = 0.90147 *p*_13_ = 0.78223 *p*_14_ = 0.60236 *p*_15_ = 0.36732 *p*_16_ = 0.12557
3rd	*A*_14_ = 0.00060	*p*_14_ = 0.99998	*A*_17_ = 0.03134 *A*_18_ = 0.01880 *A*_19_ = 0.01668 *A*_20_ = 0.01495 *A*_21_ = 0.01353 *A*_22_ = 0.01233 *A*_23_ = 0.01125 *A*_24_ = 0.01025 *A*_25_ = 0.00926 *A*_26_ = 0.00820 *A*_27_ = 0.00694 *A*_28_ = 0.00533	*p*_17_ = 0.99991 *p*_18_ = 0.99735 *p*_19_ = 0.98646 *p*_20_ = 0.96009 *p*_21_ = 0.91205 *p*_22_ = 0.83801 *p*_23_ = 0.73622 *p*_24_ = 0.60828 *p*_25_ = 0.46002 *p*_26_ = 0.30292 *p*_27_ = 0.15589 *p*_28_ = 0.04538
4th	*A*_15_ = 0.00122	*p*_15_ = 1.00000	*A*_29_ = 0.01415	*P*_29_ = 1.00000

**Figure 1:**
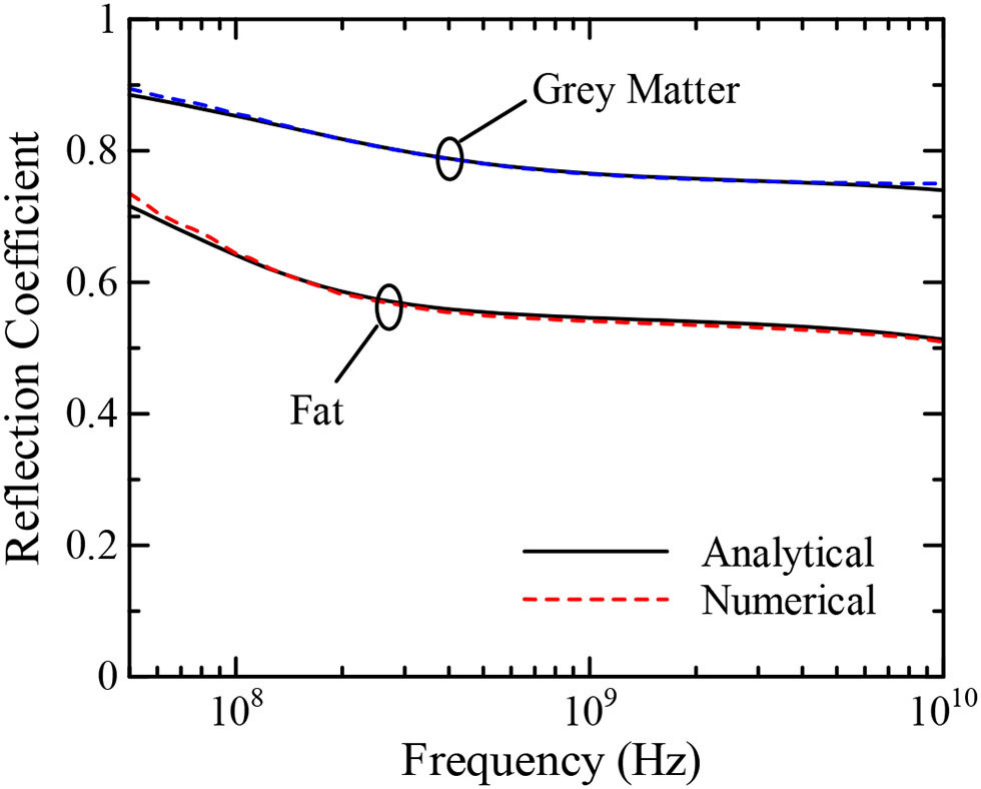
Reflection coefficients of “Fat” and “Gray Matter”.

To demonstrate the validity of the update coefficients, we calculate the reflection coefficients from each biological medium by one-dimensional FDTD simulation using the model shown as Figure 4 in Chakarothai et al. (26) and compare their values with those obtained from the EM theory. The analysis model is half filled with biological tissues and truncated with perfectly matched layers in order to absorb the outgoing wave. Numerical results using a time step interval of 1.668 ps and a resolution of 0.5 mm are shown in Figure 1. The reflection coefficients are analytically calculated using $\Gamma =|1-\sqrt{\varepsilon_{m}}|/|1+\sqrt{\varepsilon_{m}}|,$ where ε_*m*_ is the complex relative permittivity expressed by Equation (1).

As shown in Figure 1, the reflection coefficients of “Fat” and “Gray Matter” obtained via numerical simulations are within 2% of those obtained by the analytical method over a broad frequency range between 50 MHz and 10 GHz, demonstrating the validity of the update coefficients and our numerical approach. Update coefficients for the other type of biological tissues can also be calculated straightforwardly using the procedures described above. Note that when we change the time step interval, we also need to recalculate the update coefficients.

## Transmission Characteristics of EM Pulses Into Biological Bodies

### Multilayer Model

**Figure 2:**
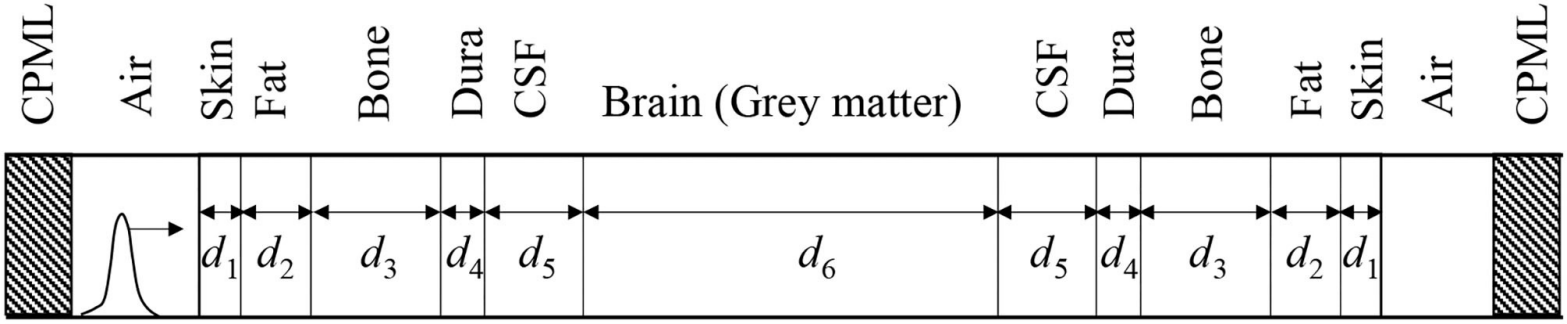
Multilayer model consisting of six biological tissues.

Figure 2 shows a multilayer model mimicking a human head, which comprises six biological tissues, similar to those used in the literature (29, 30). Table 3 indicates the thicknesses of biological tissues used in the analysis model and the number of the update coefficients for each biological tissue. The total size of the multilayer model is 180 mm. These coefficients are obtained by applying the FILT and the Prony method with a time step interval of Δ*t* = 1.668 ps. The resolution and the total number of cells used in our analysis model are 0.5 mm and 5,000, respectively. CPMLs with eight layers are utilized on both sides of the analysis domain to absorb the outgoing EM waves. The total number of time steps is 100,000. The incident electric field is given by a Gaussian pulse expressed as

(11) $$\begin{array}{l} E^{inc}\left(t\right)=\exp\left(-{\left(\frac{t-T_{0}}{a_{0}}\right)}^{2}\right)u\left(t\right), \end{array}$$

where *T*_0_ = 0.250 ns, *a*_0_ = 0.0633 ns, and *u*(*t*) is the unit step function, which is applied from the air region on the left side as shown in Figure 2. The applied Gaussian pulse contains frequency components from dc to approximately 9.3 GHz, where the power of the pulse decreases 1,000-fold from its maximum value. Number of sampling points used in fast Fourier transform to obtain the reflection coefficients and transmission characteristics is 120,000. Zero padding is used after 100,000 sampling data.

**Table 3:** Tissue density and thickness of each tissue layer.

**Tissue name**	**Thickness (mm)**	**Number of coefficients**
Skin (Wet)	*d*_1_ = 1.0	21
Fat	*d*_2_ = 1.5	15
Bone	*d*_3_ = 4.0	14
Dura	*d*_4_ = 1.0	16
CSF	*d*_5_ = 3.0	9
Brain (Gray Matter)	*d*_6_ = 159	29

**Figure 3:**
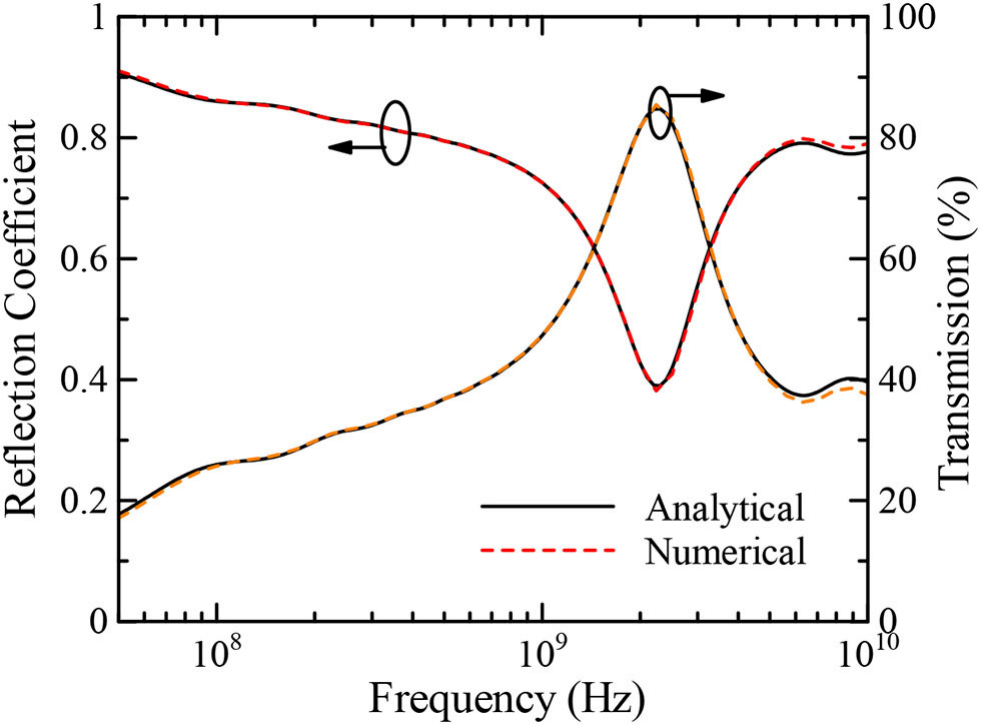
Reflection coefficient and transmission of multilayer mimicking a human head model.

Figure 3 indicates the reflection coefficient and the transmission of the multilayer model as a percentage obtained by the FDTD and analytical methods from 50 MHz to 10 GHz. The transmission, indicating the power transmitting into a biological tissue layer, is calculated using the following equation:

(12) $$\begin{array}{l} P^{t}\left(\omega \right)=\left(1-|\Gamma {|}^{2}\right)\times 100\;\left[\%\right], \end{array}$$

where Γ is the reflection coefficient. It is shown that the numerical and analytical results are in good agreement, demonstrating the validity of our proposed FDTD method again. Note also that the broadband results are numerically obtained in the time domain by a single FDTD run and transformed to those in the frequency domain via the fast Fourier transform. From the results, it can be seen that the reflection coefficient decreases with increasing frequency and reaches a minimum value of 0.38 at ~2 GHz, while the transmission exhibits peak at 2 GHz. From Figure 3, more than 80% of the incident power penetrates into the multilayer model at the maximum transmission frequency of around 2 GHz. Note that the maximum transmission frequency depends on the thicknesses of the biological tissues in the model; thus, using a different model will yield different results from those shown in this study. Next, the transmission characteristics of the EM pulse are obtained from the ratio between the receiving power at an observation point inside a biological tissue layer and the power penetrating into the model from the leftmost boundary of the skin layer.

**Figure 4:**
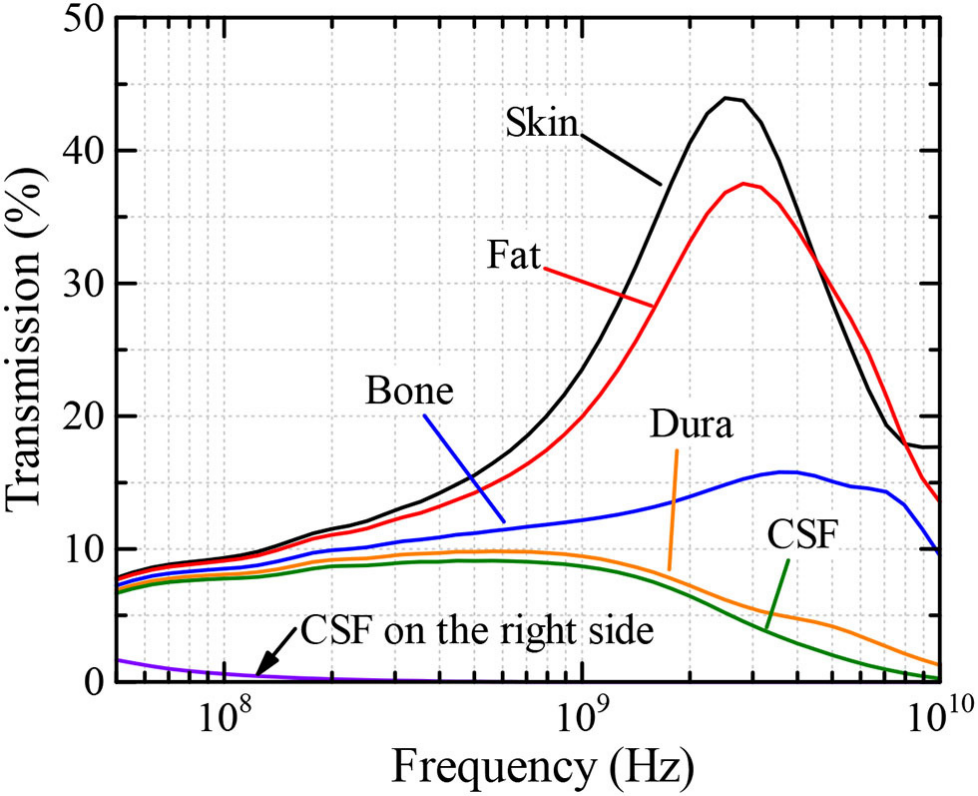
Transmission characteristics at observation points inside biological tissue layers.

Figure 4 indicates the transmission characteristics at the center of each layer in the left half of the analysis model as a percentage. From the figure, we observe a peak of the transmitted power in the skin at around 2.5 GHz. This peak shifts to a higher frequency with decreasing maximum value when the power penetrates into the subsequent layer, which is the “Bone” layer in this case. It is also shown that when the observation point is located inside the CSF layer, the transmission characteristics of this multilayer model are almost flat in the range between 300 and 800 MHz, having a maximum at ~500 MHz. In addition, when the frequency is larger than 1 GHz, most of the power is absorbed at the superficial layers before reaching the CSF layer and, therefore, the transmitted power monotonically decreases with increasing frequency in this region, except between 5 and 8 GHz, where the transmissions in the “Fat” layer is greater than that in the “Skin” layer. This may be due to a small loss in the “Bone,” compared to that of “Skin,” and multiple reflections occurring between the Skin–Fat and Fat–Bone boundaries that create a local maximum. Note that only a small proportion of the power reaches the other side of the analysis region. For example, as shown in Figure 4, the transmission power that reaches the CSF layer on the right side of the analysis model is <2% of the total transmission power and is concentrated at lower frequencies of below 100 MHz.

### Dosimetry of Various Human Bodies Exposed to EM Pulses

**Figure 5:**
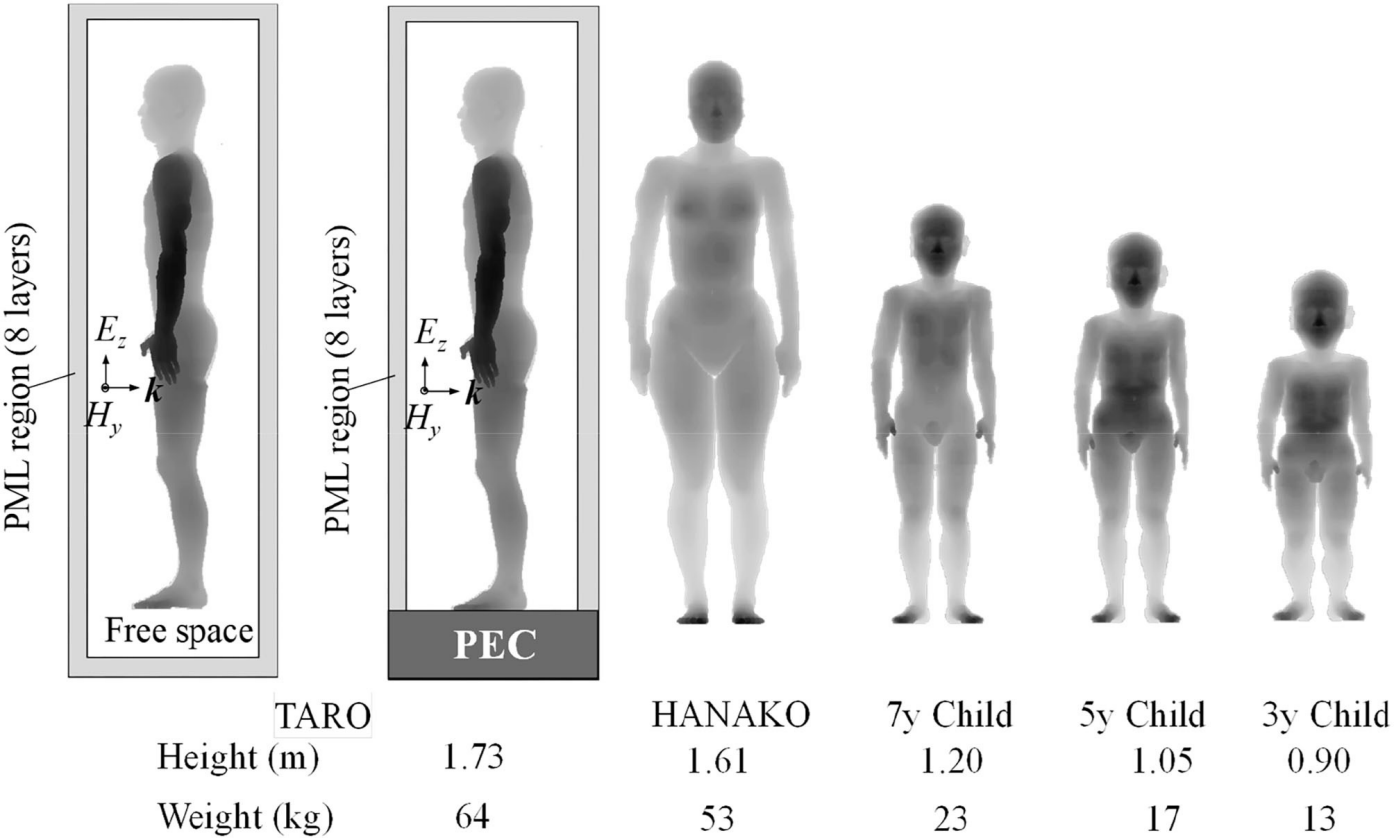
Numerical human models used in simulations. Shading color indicates the distance from the front of analysis domain. Darker for smaller distance.

As discussed in the previous subsection, the transmission into the multilayer mimicking a human head model shows a high value in the frequency range between 300 and 800 MHz at the deep tissues such as “Dura” and “CSF” and decreases above a frequency of 1 GHz due to the superficial absorption of EM energy. Therefore, our target for numerical dosimetry is an EM pulse having broad frequency components below 1 GHz. As shown in Figure 5, numerical human models of anatomical adult male (TARO), adult female (HANAKO), 7-, 5-, and 3-year child models, which were developed by National Institute of Information and Communications Technology, Japan, are chosen as our targets for numerical dosimetry (31, 32). They contain 51 different biological tissues and have a spatial resolution of 2 mm. Figure 5 also shows exposure situations with an ungrounded model (free-space model) and a grounded model standing on a ground plane made of the perfectly electric conductor (PEC). The height and weight for each numerical human model are also indicated in Figure 5. In accordance with the Courant condition, the time step interval is determined as 3.85 ps. The time step interval used here is different from that used in subsection Multilayer Model; thus, we need to recalculate the update coefficients for all biological tissues used in the numerical simulations.

The update coefficients are derived by applying the FILT and the Prony method as described above. The time step interval used to normalize the relaxation time in the Cole–Cole function is then set to 3.85 ps, the same as that used in the numerical simulations. After calculating the update coefficients, we validate them by performing one-dimensional simulations and computing the reflection coefficients. We have found that the reflection coefficients of all biological media having a permittivity function characterized by the Cole–Cole model match those obtained from the EM theory with a small difference of <2% in the frequency range between 10 MHz and 2 GHz, demonstrating the validity of the determined update coefficients used in FDTD simulations (33). The required number of terms of the update coefficients is different for each tissue, with the maximum number of *N* = 50 for “Infiltrated Cancellous Bone Barrow.” The reflection coefficients show values ranging from 0.70 to 0.90 at 10 MHz, which decrease to 0.40–0.80 at 2 GHz. The biological tissue with the lowest and highest reflection coefficients are “Non-infiltrated Bone Marrow” and “Cerebro-Spinal Fluid (CSF),” respectively. This is attributed to the fact that CSF has a higher conductivity than other tissues.

An incident electric field polarized in the *z*-direction, having the same Gaussian waveform with *T*_0_ = 0.385 ns and *a*_0_ = 0.146 ns impinges on each model from the front. The power of the incident EM pulse decreases by half at ~1.3 GHz. An EM plane wave has a polarization axis parallel with the human body axis. The amplitude of the incident electric field is 1 V/m. The calculation of SA can be carried out until the pulse strength decreased to almost zero at a specific time step. The calculation time and memory usage for each model are tabulated in Table 4. The calculation time also includes the computation time for an on-the-fly Fourier transform to obtain the electric field distributions inside the human models for use in deriving the whole-body average SAR at 24 different frequencies from 10 MHz to 1 GHz within a single FDTD run. Numerical simulations involving human models are carried out on a single calculation node (Intel Xeon E5-2680v4 @ 2.4 GHz, 256 GB memory) of TSUBAME3.0 supercomputer at Tokyo Institute of Technology, Japan, with 28 parallel threads. The total number of time steps is 10,000 steps. For our cases, total electric energy inside the human model was < -50 dB after 10,000 step, compared to its maximum during numerical simulations. The total size of the analysis region including perfectly matched layers (PMLs) are also indicated in the table.

**Table 4:** Calculation time and memory usage for each model.

**Model**	**Memory usage (GBytes)**	**Size of analysis region (cells)**	**Calculation time (s)**	**Calculation time with constant permittivity at a frequency (s)**
Male (TARO)	88.6	200 × 360 × 906	45,090 (12 h 31 min)	14,767 (4 h 6 min)
Female (HANAKO)	81.7	200 × 360 × 844	34,124 (9 h 29 min)	13,547 (3 h 46 min)
7-year child	29.2	146 × 225 × 645	11,598 (3 h 13 min)	4,677 (1 h 18 min)
5-year child	20.8	136 × 196 × 560	11,067 (3 h 4 min)	2,942 (49 min)
3-year child	16.6	132 × 187 × 481	9,126 (2 h 32 min)	2,795 (47 min)

**Figure 6:**
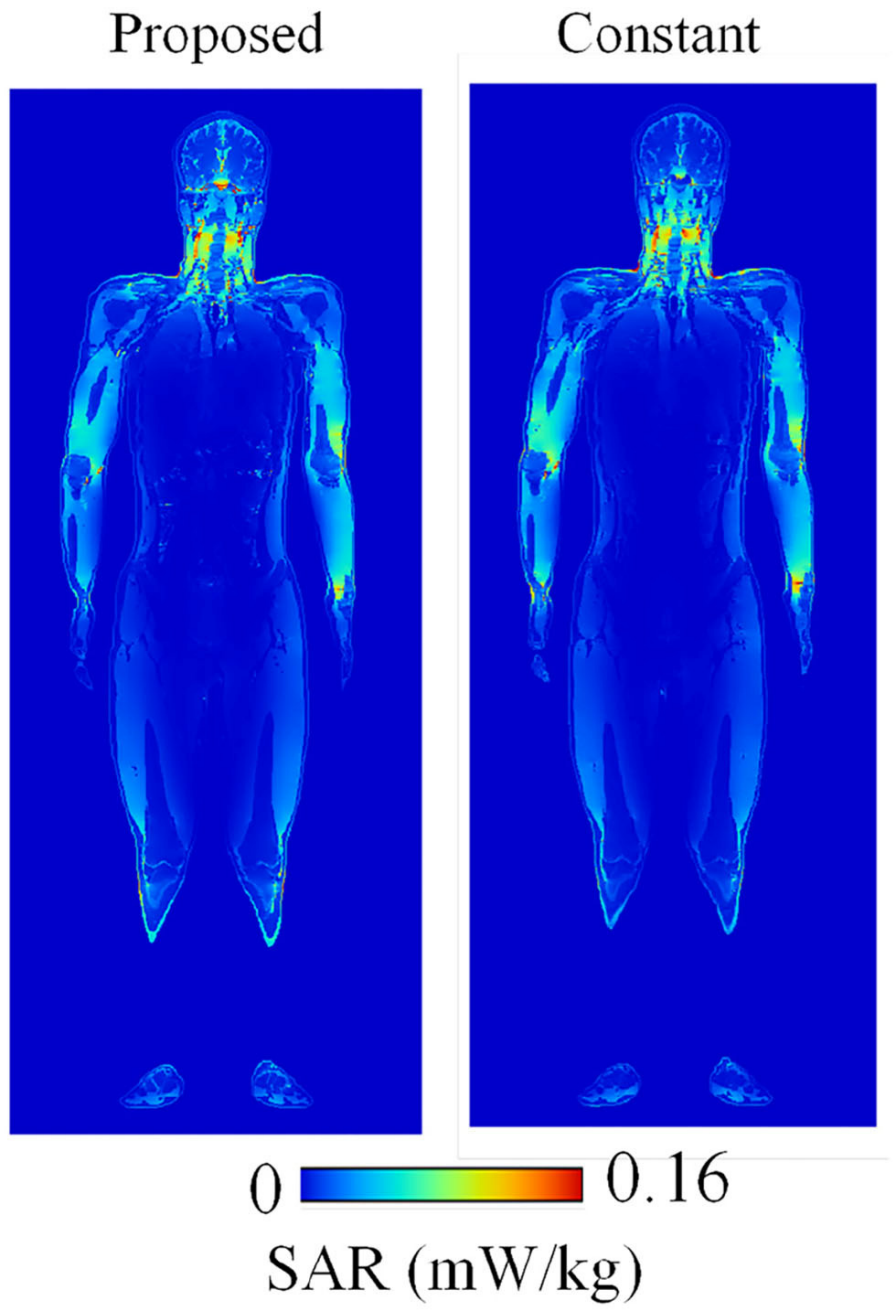
SAR distribution at 500 MHz calculated using the proposed FDTD method and that using the conventional FDTD method with constant dielectric properties.

**Figure 7:**
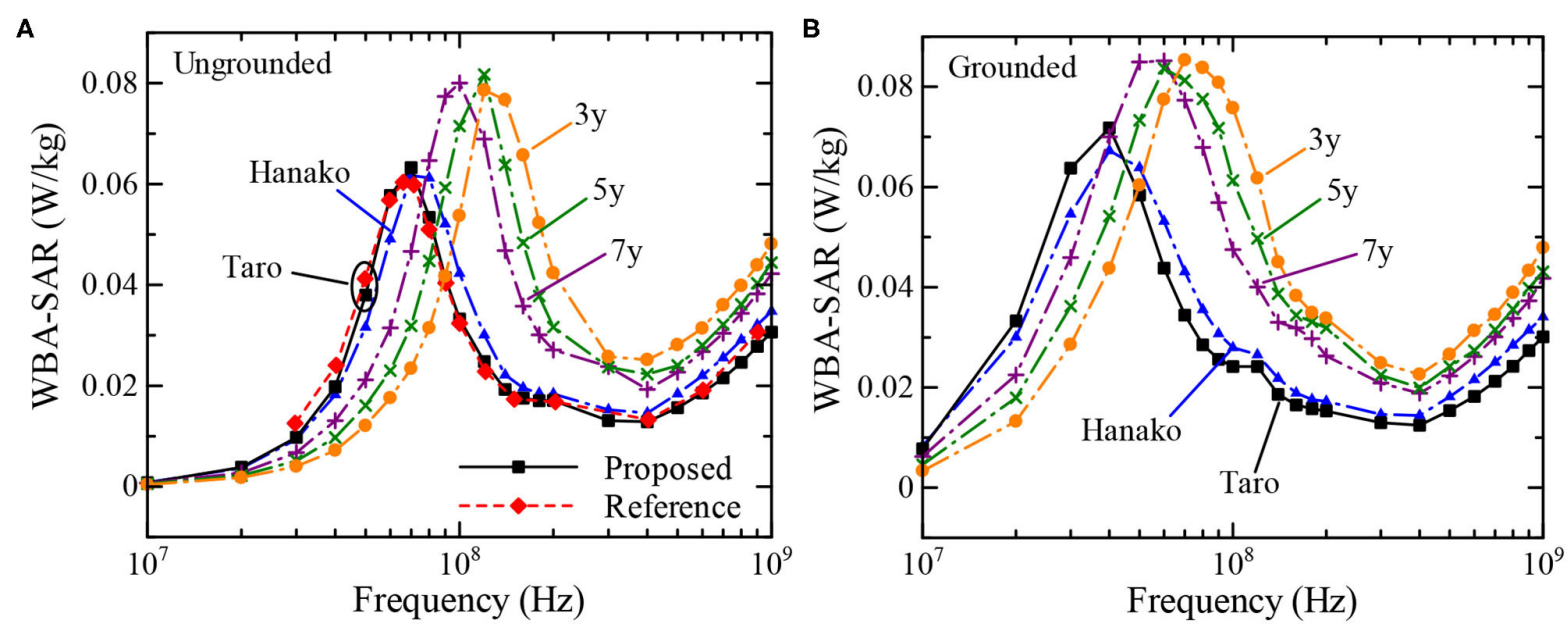
Whole-body average SAR over a frequency range between 10 MHz to 1 GHz, calculated using the proposed FDTD method for the ungrounded and grounded cases. **(A)** Ungrounded cases. **(B)** Grounded cases.

Figure 6 shows the SAR distribution inside the TARO model illuminated by a planewave with a 1 V/m electric field at 200 MHz. The results are obtained by using the proposed (FD)^2^TD approach and using constant dielectric properties for comparison. Both distributions are well-matched, demonstrating the validity of the (FD)^2^TD method. Figures 7A,B indicate the whole-body-average specific absorption rate (WBA-SAR) of various numerical human models for the ungrounded and grounded conditions, respectively. Note that our numerical results are obtained by a single run of FDTD computation for each model. The WBA-SAR at each frequency is calculated using an on-the-fly Fourier transform during the FDTD run. It is shown that the results obtained by the proposed method are in good agreement with those derived in the literature, which are also shown as a red dashed line in the figure (34), again demonstrating the validity of our proposed method. Note that the results in Figure 7 are normalized by the limits of the incident power density prescribed by the ICNIRP guidelines at each frequency. As indicated in Table 4, our proposed method requires almost three time longer than the conventional one using constant permittivity at a frequency, however, the conventional method can only provide WBA-SAR at only one frequency per FDTD computation and number of time steps required to obtain the converged solution is different for each frequency (35). Meanwhile, the proposed method requires only a single FDTD run to determine the solutions at 24 analysis frequencies. Although the proposed method requires more memory usage, in consequence, it is shown to be very computationally efficient. As number of frequency points increases, efficiency of the proposed method increases at the expense of using more memory. From Figure 7, the SAR peak is found at around 70 MHz for the ungrounded TARO model, which corresponds to the whole-body resonance and the peak frequency increases for the shorter HANAKO, 7-, 5-, and 3-y-child models. For grounded conditions, the SAR peaks occur at 40 and 70 MHz for the adult and 3-y-child models, respectively. These results are in good agreement with those indicated in the literature (32, 34).

**Figure 8:**
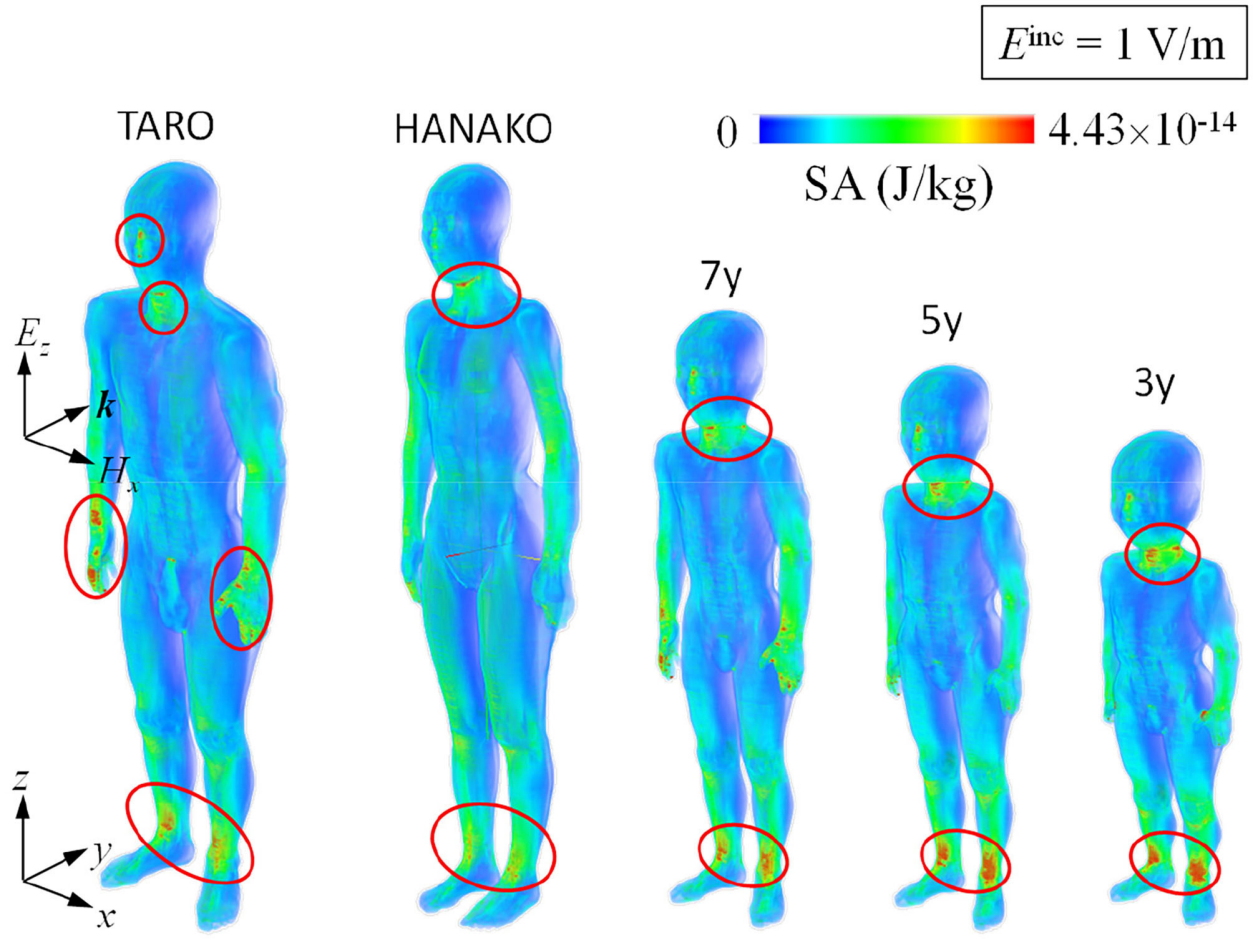
SA distribution for one pulse illumination for various ungrounded human models.

**Figure 9:**
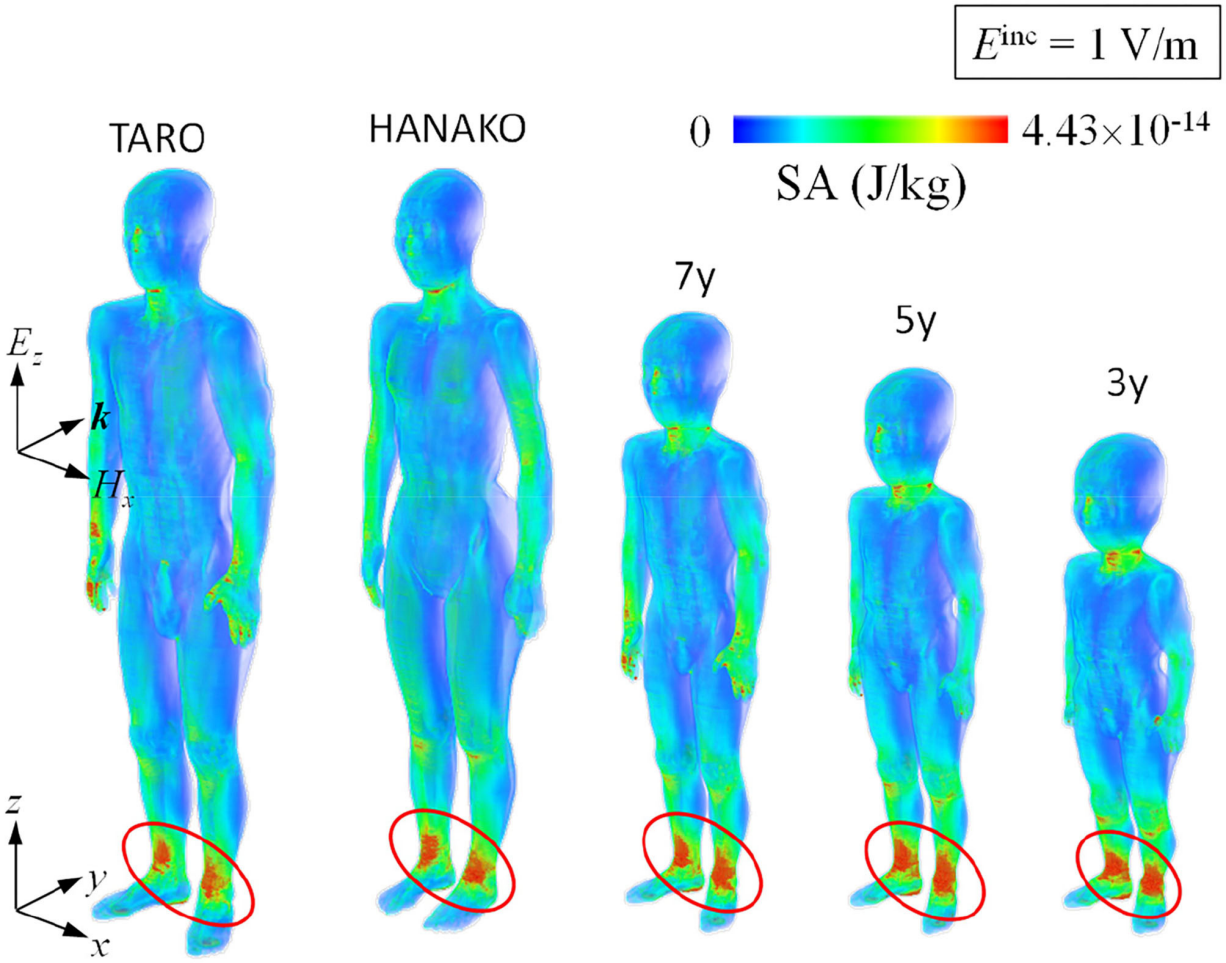
SA distribution for one pulse illumination for various grounded human models.

**Figure 10:**
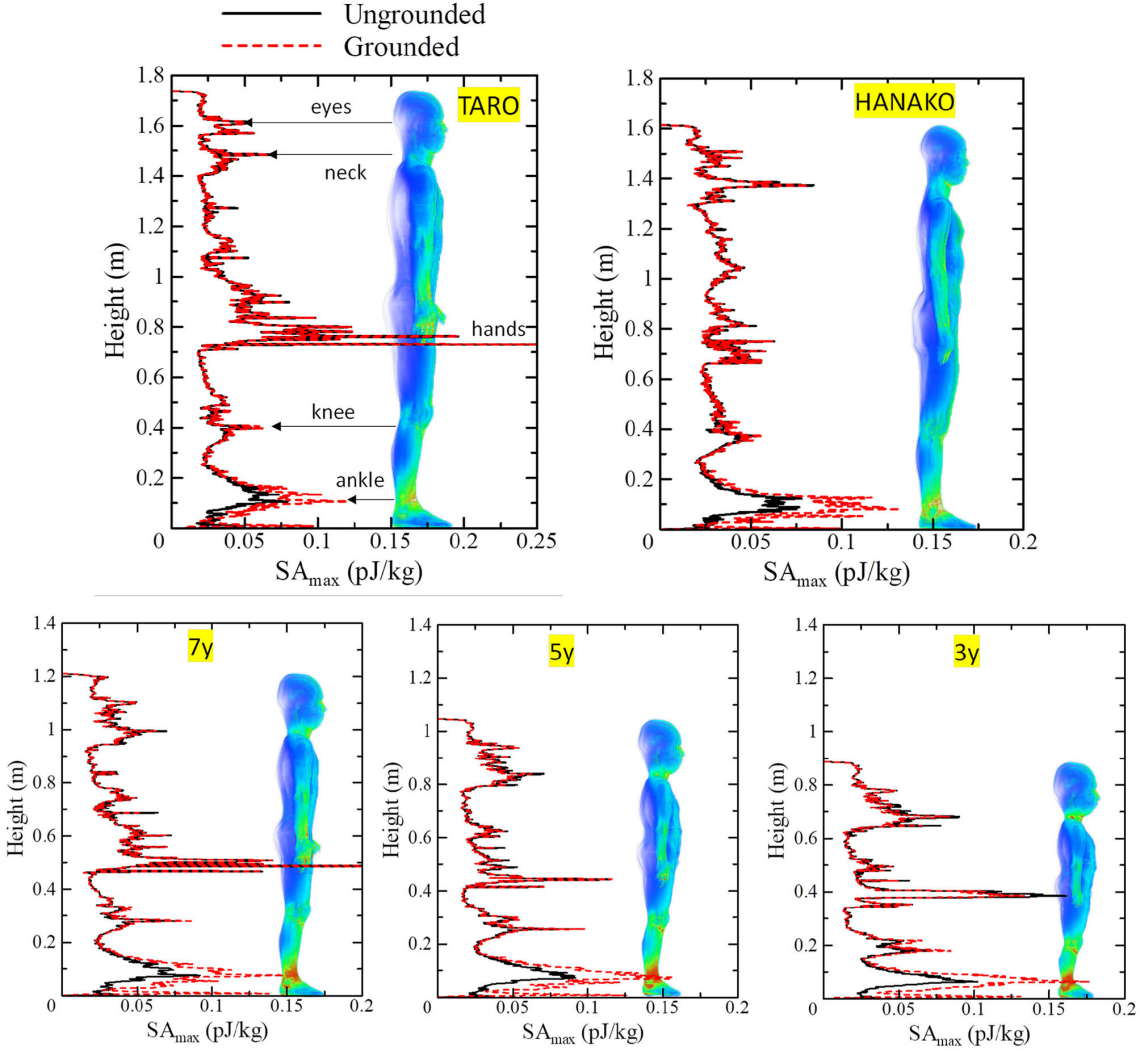
Maximum SA at each height for various human models for 1 V/m electric field strength.

Figures 8, 9 show SA distributions on the human surface for ungrounded and grounded cases, respectively. For the ungrounded cases, SA peaks appear at the ankles, wrists (hand), forearm, and neck; SA increases by 53% (adult male) to 74% (3-y-child) at the ankle, whereas SA at the other parts remain almost the same in the ungrounded cases. Figure 10 shows the layer maximum SA at each height of various human bodies when exposed to an EM pulse of 1 V/m, polarized in the z-direction. The peak SA and the SA at the ankle for various human models, which are normalized to the ICNIRP-prescribed power density limit of 2 W/m^2^, are summarized in Table 5 (10). Note that the power density limit is constant for 10–400 MHz and increases with respect to the frequency over 400 MHz. Therefore, there is no prescribed limit value of the power density for a wideband pulse such as one used in our study. However, we hereby use 2 W/m^2^ for the normalization of SA in Table 5 as it should provide conservative evaluations. The maximum SA appears at the hands for the adult male, 7-, 5-, and 3-y-child models for the ungrounded case, while it appears at the neck for the adult female model. This may be attributed to the proportion of biological tissues in the male and female human models is different since the female model contains more fat in each body part. Note that 7-, 5-, and 3-y-child models are proportionally morphed using a morphing algorithm (32). The maximum SA among five human models is 0.437 pJ/kg. Note that this SA value also depends on the waveform (which is the Gaussian pulse in our study). To reach a dose of 2 mJ/kg, as prescribed in the ICNIRP guidelines, we need to increase the field strength from 1 V/m to more than 83 kV/m or 9.14 MW/m^2^, which does not seem realistic in real life. Note that SA is obtained from the value at a voxel and it should be smaller for an average over 10 g tissues. Hence, the SA values shown in Table 5 assume a worst-case scenario. For compliance with the IEEE standards, the repetition rate of an incident pulse having a field strength of 87 kV/m must be <800 Hz or 800 pulses per second in order not to exceed the SA limit. In the grounded case, the maximum SA occurs at the ankle for the adult female, 5-, and 3-y-child while they are found at the same location (hands) in the ungrounded case for the adult male and 7-y-child. It is found that energy absorption at the ankle increases when the human body is grounded, while that at the other parts remains almost unchanged for all the models used in this study. Table 6 shows the peak 1 g-averaged and 10 g-averaged SAs normalized by a power density limit of 2 W/m^2^. It is shown that the peak 1 g-averaged and 10 g-averaged SAs are higher in the grounded cases than those in the ungrounded cases for all models. The increases in the SAs are significant in a smaller model, e.g., for the grounded 3-y-child model, the peak 1 g-averaged and 10 g-averaged SAs are ~1.67 and 1.76 than those of the ungrounded cases. These results provide the first ever demonstration that the SA distribution due to broadband EM pulse illumination can be quantitatively evaluated in detail and compared with the SA limit prescribed in international guidelines or standards. Further detailed exposure levels for different incident angles, different pulse shapes, and postures will be investigated in the future.

**Table 5:** Maximum specific energy absorption normalized by an ICNIRP-prescribed power density limit of 2 W/m^2^ for general exposures to various human model.

**Model**	**Ungrounded case**		**Grounded case**	
	**Maximum SA (nJ/kg)**	**SA at the ankle (nJ/kg)**	**Maximum SA (nJ/kg)**	**SA at the ankle (nJ/kg)**
Male (TARO)	0.401 (hand)	0.119	0.401 (hand)	0.182
Female (HANAKO)	0.127 (neck)	0.117	0.199 (ankle)	0.199
7-year child	0.437 (hand)	0.139	0.436 (hand)	0.223
5-year child	0.173 (hand)	0.138	0.237 (ankle)	0.237
3-year child	0.243 (hand)	0.154	0.267 (ankle)	0.267

**Table 6:** Peak 1 g-averaged and 10 g-averaged SA normalized by an ICNIRP-prescribed power density limit of 2 W/m^2^ for general exposures to various human model.

**Model**	**Ungrounded case**		**Grounded case**	
	**1 g-averaged SA (pJ/kg)**	**10 g-averaged SA (pJ/kg)**	**1 g-averaged SA (pJ/kg)**	**10 g-averaged SA (pJ/kg)**
Male (TARO)	0.0846	0.0536	0.1019	0.0616
Female (HANAKO)	0.0737	0.0433	0.1234	0.0632
7-year child	0.0821	0.0457	0.1310	0.0710
5-year child	0.0947	0.0507	0.1543	0.0839
3-year child	0.1013	0.0512	0.1691	0.0900

## Conclusion

We have performed numerical dosimetry on human bodies illuminated by an EM pulse from the front by using the (FD)^2^TD method, previously proposed by the authors. The method fully considers broadband characteristics of the complex relative permittivity of the biological media used in the analysis model via the application of the FILT and the Prony method. Firstly, we demonstrated the validity of the update coefficients, i.e., the residues and poles of the expression for the IIR in the z-domain, by comparing the numerical reflection coefficients with those derived from the EM theory. It was clarified that the numerical results within 2% of those obtained theoretically over a broad frequency range from 50 MHz to 10 GHz, demonstrating the validity of the proposed approach. It was also found that the transmission characteristics of the EM pulse into the CSF layer of a multilayer mimicking a human head are almost flat over a frequency range between 300 and 800 MHz and that the transmission decreases with increasing EM traveling distance from the skin boundary due to higher energy absorption at superficial biological tissues such as “Skin” and “Fat” when the frequency is higher than 1 GHz. Therefore, most of the pulse energy that penetrates into the biological body has a frequency below 1 GHz. Then, numerical dosimetry of various human models exposed to an EM pulse having a frequency component of up to ~1.3 GHz was performed. The whole-body average SAR at 24 frequencies was determined by a single run of broadband FDTD simulations. The results matched those published in the literature, demonstrating the validity and availability of the proposed FDTD method. Then the SA distribution of each numerical model was determined, and it was found that the maximum SA occurs at the hands and neck for the ungrounded model, while they appear at the hands and ankle when the model is grounded. The maximum SA value was 0.290 pJ/kg for an incident electric field strength of 1 V/m or 0.437 nJ/kg for an incident power density of 2 W/m^2^. It has been shown for the first time that, by using our proposed FDTD approach with the FILT and the Prony method, we can obtain quantitatively detailed information on SA that can be compared with the limits prescribed in international guidelines or standards.

## Data Availability Statement

The original contributions presented in the study are included in the article, further inquiries can be directed to the corresponding author.

## Author Contributions

JC designed and performed all the simulations, analyzed the data, and wrote the paper. KW and KF gave advices and revised the paper. All authors have read and agreed to the published version of the manuscript.

## Conflict of Interest

The authors declare that the research was conducted in the absence of any commercial or financial relationships that could be construed as a potential conflict of interest.

## Publisher's Note

All claims expressed in this article are solely those of the authors and do not necessarily represent those of their affiliated organizations, or those of the publisher, the editors and the reviewers. Any product that may be evaluated in this article, or claim that may be made by its manufacturer, is not guaranteed or endorsed by the publisher.
